# Strategy for early diagnosis of pancreatic cancer

**DOI:** 10.3389/fgstr.2026.1731223

**Published:** 2026-09-30

**Authors:** Ravi Kumar Sharma, Alpa Singh, Gaurav Sharma, Saurabh Dawra, Surinder Singh Rana

**Affiliations:** 1 University Institute of Biotechnology, Chandigarh University, Mohali, Punjab, India; 2 Department of Pharmacology, Central Drug Research Institute, Lucknow, India; 3 Department of Gastroenterology, Command Hospital, Udhampur, Jammu and Kashmir, India; 4 Department of Gastroenterology, Postgraduate Institute of Medical Education and Research, Chandigarh, India

**Keywords:** biomarkers, CA 19-9, early diagnosis of PDAC, new-onset diabetes mellitus, pancreatic cancer, pancreatic ductal adenocarcinoma

## Abstract

Exocrine pancreatic cancer, particularly Pancreatic Ductal Adenocarcinoma, is regarded as one of the most aggressive cancers with a poor prognosis. Its bleak outlook mainly results from its silent progression, late diagnosis, and unreliable early detection methods. Globally, PC ranks among the leading causes of cancer-related deaths, and its incidence continues to increase. Because the pancreas is situated deep within the abdomen, early symptoms such as mild abdominal pain, fatigue, or weight loss are often vague. Most patients are only diagnosed after the disease has spread beyond the point where curative treatment is possible. Although surgery provides the best chance for long-term survival, only a small number of patients qualify for it at the time of initial diagnosis. Several factors increase the risk of developing PC, including chronic pancreatitis, smoking, obesity, inherited genetic mutations (like BRCA1/2), and new-onset diabetes (NOD), which has recently gained attention as a possible early warning sign. However, widespread screening for PC isn’t yet practical because the disease is relatively uncommon and there are no highly accurate, non-invasive biomarkers. The most widely used blood test, CA 19-9, often gives unreliable results, while newer molecular approaches—like circulating tumor DNA, exosomal markers, microRNAs, and metabolomic profiles—are still being refined. Recent advances in imaging technology, artificial intelligence, and targeted screening for high-risk individuals hold promise for catching pancreatic cancer earlier. As we advance, combining multi-omics biomarkers with precision imaging may finally make earlier diagnosis—and improved survival—a reality for this devastating disease.

## 1 Introduction

Pancreatic cancer (PC), especially pancreatic ductal adenocarcinoma (PDAC), is one of the deadliest malignancies worldwide (1). Despite advances in medical research, the prognosis remains poor due to its late clinical presentation and aggressive nature (2). The five-year survival rate for PDAC remains under 12.8%, largely because most cases are diagnosed at an advanced stage (2, 3). Although early detection has the potential to improve drastically it remains a significant clinical challenge (4). According to the latest data from the Global Cancer Observatory, approximately 496,000 new cases and 466,000 deaths were reported worldwide, highlighting its extremely poor survival rate (5, 6).

Deep anatomical location of pancreas and nonspecific early symptoms such as abdominal discomfort, fatigue, and weight loss, often lead to delayed diagnosis (7). By the time symptoms prompt medical attention, most tumors have advanced beyond the stage of curative treatment. PDAC, for approximately 85-90% of PC, is highly aggressive, characterized by rapid local invasion and early distant metastasis (8). Consequently, only about 15-20% of patients are eligible for surgical resection at diagnosis (9). Notably, PDAC, which represents the predominant histopathological subtype, shows a particularly strong association with new-onset diabetes (NOD) As a results NOD has emerged as an important clinical clue warranting further evaluation for early pancreatic malignancy (10, 11).

Early diagnosis is the most effective means of improving survival in PDAC. When diagnosed at an early, localized stage, patients have a significantly better prognosis, with five-year survival rates improving from less than 10% to nearly 35-50% (12). Currently, there is no population-wide screening program for PC, mainly because the disease has a relatively low incidence and lack of reliable, non-invasive diagnostic markers (13). However, targeted screening of high-risk populations such as familial PC syndromes, genetic predispositions, or significant clinical risk factors is increasingly being adopted. For example, the International Cancer of the Pancreas Screening (CAPS) consortium recommends surveillance of high-risk individuals using advanced imaging techniques such as magnetic resonance imaging (MRI) and endoscopic ultrasound (EUS) to detect early, asymptomatic lesions of PDAC (14). Despite the well-established benefits of early detection, several key limitations hinder the timely diagnosis of PDAC (15). The most widely used serum marker, Carbohydrate Antigen 19-9 (CA 19-9), has limited sensitivity and specificity, especially in early-stage disease (16). Its levels can be elevated in benign conditions such as pancreatitis and biliary obstruction, and approximately 5–10% of individuals do not express it due to genetic variations (17). Although molecular diagnostics focusing on circulating tumor DNA (ctDNA), exosomes, microRNAs, and metabolomics signatures show promise, these tools remain experimental and are not yet validated for routine clinical application (18, 19). Current Imaging modalities lack sufficient sensitivity for early stage PDAC detection, and the low disease prevalence renders universal screening impractical, emphasizing the need for refined risk stratification strategies (20). Another challenge lies in the detection and management of precursor lesions such as pancreatic intraepithelial neoplasia (PanIN), intraductal papillary mucinous neoplasm (IPMN), and mucinous cystic neoplasm (MCN). These lesions have the potential to progress to invasive cancer but are often difficult to detect non-invasively. Moreover, the unpredictable timeline of progression complicates clinical decision-making regarding surveillance and surgical intervention (21).Existing diagnostic modalities, including serum CA 19–9 and conventional imaging techniques, lack adequate sensitivity and specificity for detecting early-stage disease. Although numerous studies have investigated emerging approaches such as liquid biopsy, molecular biomarkers, and advanced imaging technologies, most remain in exploratory phase and have not yet been translated into routine clinical practice. Consequently, there is a critical need to develop and refine novel diagnostic strategies capable of identifying PDAC (22). This review critically evaluates current and emerging strategies for early diagnosis of PDAC, with emphasis on risk stratification, biomarkers, imaging modalities, and surveillance of high-risk populations, while highlighting key challenges and future directions in the field.

## 2 Identification of high-risk individuals

### 2.1 Family history & familial pancreatic cancer

Family history is a well-established risk factor for PC, particularly PDAC, and plays a crucial role in identifying individuals who may benefit from early detection strategies (23). Approximately 10–15 percent of PDAC cases occur in individuals with a positive family history of the disease or an identifiable hereditary predisposition syndrome, underscoring the contribution of inherited susceptibility to disease development (24, 25). Data from the PC Case–Control Consortium estimate heritability of PDAC to be approximately 21.2 percent, highlighting the strong genetic component of familial aggregation (26). FPC is defined by the presence of two or more first-degree relatives diagnosed with PC and accounts for approximately 5–10 percent of all PDAC cases (27) Individuals from FPC families carry a substantially elevated risk compared with the general population. The risk roughly doubles with one affected first-degree relative, increases sir-fold with two affected relatives, and may exceed a 30-fold increase when three or more first-degree relatives are affected, making family history a critical criterion for surveillance (28). Several hereditary cancer syndromes are associated with an elevated risk of PDAC (29) (**Figure 1**).

**Figure 1:**
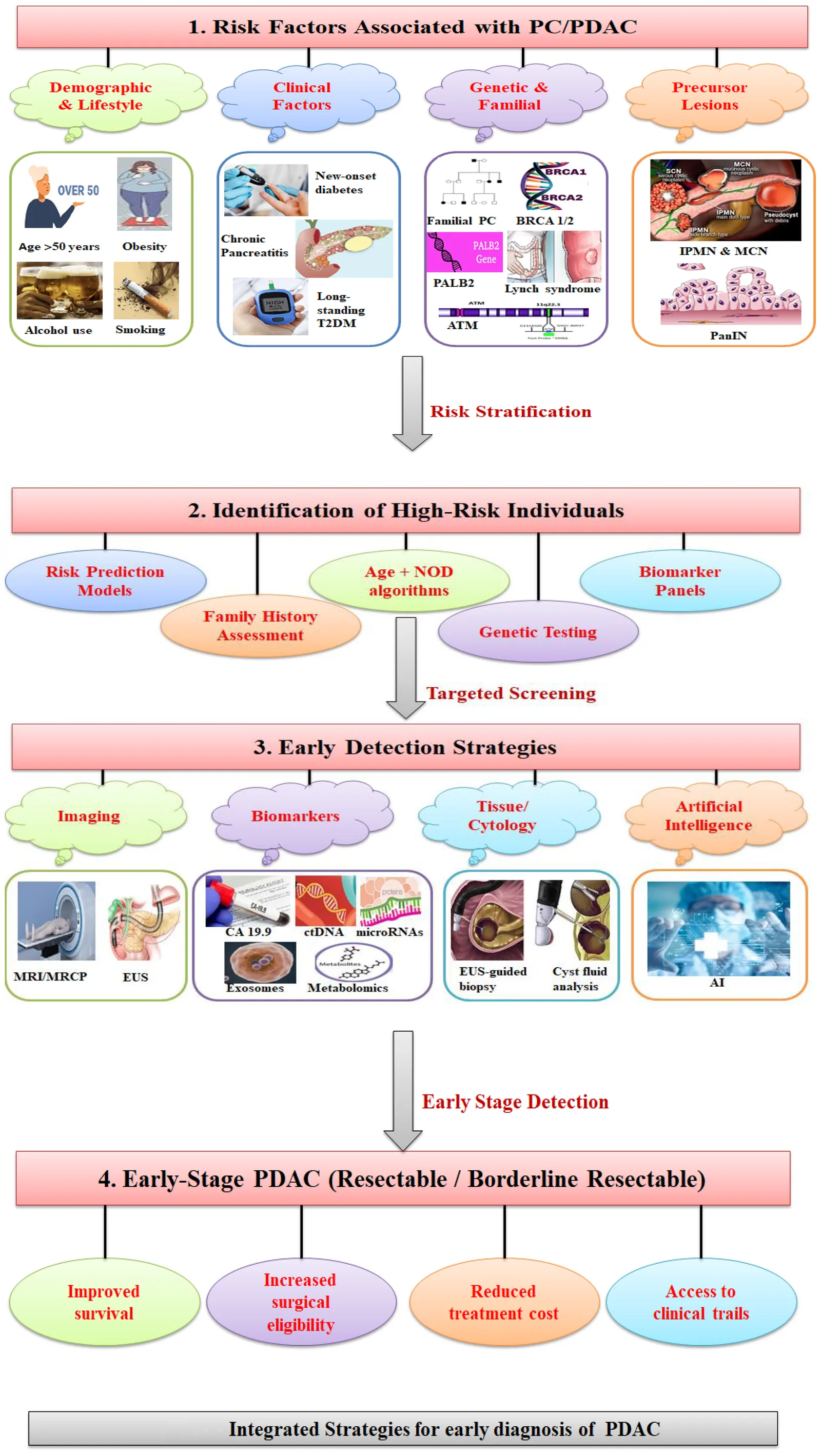
Integrated strategies for early diagnosis of PDAC.

Mutations in BRCA1 and BRCA2 commonly associated with hereditary breast and ovarian cancer syndrome may have a 3-10-fold higher risk of developing PDAC (30). On the other hand, Lynch syndrome, caused by mutations in mismatched repair genes such as MLH1 and MSH2, also significantly increases the likelihood of PC (31). Other syndromes, such as familial atypical multiple mole melanoma (FAMMM) related to CDKN2A, Peutz-Jeghers syndrome (PJS) associated with STK11/LKB1, and hereditary pancreatitis caused by mutations in the PRSS1 gene, may increase the risk up to 50-fold (32). Based on these familial genetic risk profile, HRIs are increasingly enrolled in structures surveillance programs aimed at early detection. Current guidelines from CAPS consortium recommended surveillance using MRI/MRCP and/or EUS, typically starting at age 50 or 10 years younger than the youngest affected family member. Evidence from CAPS studies demonstrate that such targeted surveillance can detect precancerous lesions and early-stage PDAC, thereby increasing the likelihood curative surgical intervention and improved survival outcomes (33, 34).

### 2.2 Genetic predisposition syndrome

A significant subset, approximately 5–10% of, particularly PDAC arises in the context of inherited genetic Syndromes. Germline mutations in tumor suppressor or DNA repair genes confer and early detection strategies. Among these, BRCA1 and BRCA2 are the two most commonly known genes associated with FPC (36) (**Figure 1**). These tumor suppressor genes often linked to hereditary breast and ovarian cancer syndrome, BRCA2 mutations particularly have a 5 to 10-fold greater PDAC risk, which is frequently detected in FPC cases; however, BRCA1 is comparatively associated to a lesser extent (27, 37). Therefore, the occurrence of these mutations indicates earlier and more frequent screening, particularly in families with multiple cancer types. The CDKN2A gene is linked to FAMMM Syndrome, represent one of the strongest genetic risk factors for PDAC. Germline CDKN2A mutations may increase PDAC risk up to 30 to 40 times higher, justifying prioritization of these individuals for early detection using imaging modalities such as EUS, MRI/MRCP (38). Another high-penetrance syndrome is PJS, caused by mutation in STK11/LKB1. PJS is one of the most significant lifelong risk of PDAC, and surveillance is often initiated in a younger age. Sometimes as early as the third and fourth decade, irrespective of family history (39). Mutations in PALB2, a “partner and localizer of BRCA2”, involved in DNA repair via homologous recombination, are associated with a slightly elevated risk of PDAC [ (40, 41). markedly increased lifetime PDAC risk, making affected individuals (35) targets for surveillance Although they are less effective than the BRCA2 or CDKN2A, their mutations are frequently observed in families with breast and ovarian cancer requires consideration in early detection techniques (42). PRSS1 gene mutations are caused by hereditary pancreatitis, is another important genetic condition predisposing to PDAC (43). Chronic pancreatic inflammation in these individuals are expected to have a 40-55% lifelong risk of PDAC, especially among long-term smokers, emphasizing the need for early and advanced surveillance (44). The ATM gene is another emerging risk factor, involved in DNA damage response, are increasingly recognized in both familial and sporadic PDAC (45). Though, severity of risk is lesser compared to high-penetrance genes, their inclusion in genetic evaluation can be beneficial for risk stratification and screening decisions (46). Finally, Lynch syndrome, caused by mutations in mismatch repair genes (MLH1, MSH2, MSH6, and PMS2), is association with an approximately 6–8 times increased risk of PDAC (47). Therefore, affected individuals may not arrive with a family history of PC specifically, but their genetic profiles still requires surveillance if additional risk factors are present (48).

## 3 Clinically relevant risk factors for early detection

### 3.1 New-onset diabetes mellitus

NOD over the age of 50 has been identified as an important diagnostic marker and risk factor for early PDAC detection (49), Epidemiological studies have shown that people who develop diabetes over the age of 50 without a known causal factor (such as obesity or family history) are 6 to 8 times at higher risk of developing PDAC than the general population (50). In fact, in certain people, NOD may be an early sign of an underlying, asymptomatic pancreatic tumor (51). Recent evidence has refined the diagnostic utility of NOD through clinical risk stratification. A 2022 systematic review and meta-analysis identified clinical features among new-onset diabetics that further stratify cancer risk: a family history of PC (OR 3.78, 95% CI 2.03–7.05), history of pancreatitis (OR 5.66, 95% CI 2.75–11.66), and rapid weight loss (OR 2.49, 95% CI 1.47–4.22) were among the strongest predictors of an underlying malignancy within one year of diabetes diagnosis (49). Early recognition of these high-risk profiles may facilitate timely pancreatic imaging and improve diagnostic yield. To operationalize NOD as a diagnostic tool, risk prediction models such as the Enriching New-Onset Diabetes for Pancreatic Cancer (ENDPAC) score includes age, weight loss, and changes in glycemic control to identify high-risk patients for imaging-based screening (52, 53). This current approach used to detect tumors at an earlier, possibly resectable stage, when treatments are more efficient. Prospective screening studies further support the feasibility of this approach. In structured program fast-tracking individuals aged ≥50 years with new-onset or rapidly deteriorating diabetes to MRI/MRCP based surveillance, PDAC detection rate of approximately 0.9 percent have been reported, with screen-detected cases diagnosed at earlier stage than typically observed in sporadic presentations (4, 54). In one longitudinal cohort, annual imaging combined with biomarker assessment identified early stage PDAC without major safety concerns (54). Collectively, these findings, indicate that NOD represents a practical, non-invasive entry point for risk stratification in sporadic PDAC, particularly in individuals without known hereditary predisposition. Ongoing efforts now focus on integrating NOD with imaging and emerging biomarkers to enhance positive predictive value and refine early detection algorithms (55).

### 3.2 Chronic pancreatitis

CP is a well-known risk factor for PDAC (56) Long-standing pancreatic inflammation is associated with progressive fibrosis and tissue remodeling, creating a biological context in which malignant transformation may occur (56). Epidemiological studies indicate that people with CP have a considerably greater risk of PDAC, estimated to be up to 10–16 times higher than the general population (57, 58). The risk is even more significant in hereditary pancreatitis, which is caused by mutations in the PRSS1 gene, where lifetime PDAC risk may increase by 40-50% (59). Duration of disease is a critical modifier, with the highest relative risk observed within the first few years after CP diagnosis (60). A meta-analysis of observational studies reported a pooled effect estimate of 16.16 (95% CI 12.59–20.73) for cancers diagnosed within two years of CP onset, with risk remaining nearly eightfold at five years (EE 7.90; 95% CI 4.26–14.66) and over three-fold after nine years (EE 3.53; 95% CI 1.69–7.38) (56). Additional clinical features including, concomitant diabetes, exocrine insufficiency, and low body mass index further identify CP patients at highest risk (61).From an early diagnostic perspective, CP presents a unique clinical challenge, as symptoms of chronic inflammation often overlap with those of early PDAC, leading to delay in diagnosis of malignancy. While CP markedly increases PDAC risk, routine surveillance of all CP patients is not currently recommended, due to insufficient evidence that population-wide screening improves outcomes. The American Academy of Family Physicians notes that, despite the higher incidence, it is not known whether screening asymptomatic CP patients with imaging or biomarkers reduces mortality, and instead recommends focused evaluation—such as MRI or EUS—for new symptoms like weight loss or jaundice rather than blanket surveillance (62). Likewise, the American Gastroenterological Association advises reserving formal screening for hereditary pancreatitis beginning at age 40–50, and cautions that non-genetic CP patients should only undergo surveillance within research protocols or when clinical changes raise suspicion of neoplasia (63). Consistent with these specialty guidelines, the US Preventive Services Task Force reaffirms a recommendation against routine PC screening in asymptomatic adults, emphasizing the current lack of evidence that such programs confer net benefit even in high-risk groups (64).

### 3.3 Pancreatic cystic lesions

PCLs represent an increasingly recognized risk-enriched state for PDAC, particularly as their detection has risen with widespread use of cross-sectional imaging (65). Among various cystic lesions intraductal IPMNsMCNs are the most clinically relevant premalignant entities and constitute important targets for early diagnosis and cancer prevention strategies (66). Lesions involving the Main duct and mixed-type IPMNs had the highest risk for developing malignant tumors, with studies indicating a 50-60% chance of progression to high-grade dysplasia or invasive malignancy (67). While branch duct IPMNs have a lesser risk, particularly if they exhibit to characteristics such as cysts larger than 3 cm, mural nodules, or main pancreatic duct dilatation, substantially increases the likelihood of malignancy and warrants closer surveillance or intervention (68). Although MCNs are less common than IPMNs, they can be malignant, especially if the lesion is large or has nodular components (69). Therefore, surgical resection of MCNs is frequently recommended in medically fit patients, reflecting their role in preventing progression in PDAC due to the risk of progression to invasive carcinoma (70). Therefore, PCLs serve as an essential marker for early diagnosis of PC. Since these lesions are incidentally found during imaging enabling risk stratification before the onset of invasive disease. Current management strategies rely on MRI/MRCP and EUS for longitudinal assessment, with resection decisions guided by radiologic features predictive of malignancy Additionally, molecular analysis of a cystic fluid, including detection of KRAS and GNAS mutations, may also help in identifying high-risk lesions, although its clinical integration remains investigational (71).

## 4 Limitations of current diagnostic approaches

Early detection of PDAC remains a major challenge, particularly in high-risk individuals with familial predisposition or pathogenic germline variants. Imaging plays a pivotal role in risk stratification, surveillance, and clinical decision-making (72). Contemporary strategies emphasize a tiered approach, integrating non-invasive modalities for baseline surveillance with problem-solving and staging tool when indicated (22).

### 4.1 Imaging modalities

**MRI**, in conjunction with MRCP, represents the preferred imaging strategy for early diagnosis and surveillance of PDAC, especially in individuals with precursor lesions or elevated risk (73). MRI/MRCP offers high-resolution images of soft tissues without resorting to ionizing radiation and can be repeatedly utilized for surveillance protocols (74).It is particularly valuable in detecting subtle irregularities of the ducts, including dilatation, strictures, or communication with cystic lesions, which are relevant in the assessment of IPMNs and other pancreatic cysts with malignant potential (75). MRI/MRCP is important for risk stratification assessing for mural nodules, size of the cysts, involvement of the main duct, and follow-up changes to decide which cysts should be surgically resected and which should be followed up (76). As a result, MRI/MRCP forms the foundation of most contemporary pancreatic surveillance protocols. Current surveillance practices in patients with known pancreatic cysts or high genetic risk (e.g., BRCA mutations, FPC) tend to suggest annual or biennial MRI/MRCP (77). These are endorsed by societies like the American College of Gastroenterology (ACG) and CAPS Consortium and view MRI/MRCP as the method of choice over computed tomography (CT) based on improved contrast resolution and safety profile (78) EUS with Fine-needle aspiration (FNA) is a mainstay in the imaging and surveillance plan for PC, especially for the assessment of small lesions and indeterminate cystic or solid masses (79). In combination with FNA, EUS facilitates both diagnostic imaging and tissue sampling, which is critical for cytologic and molecular analysis (80). EUS is especially useful in identifying small pancreatic tumors (<2 cm), which tend to be missed by other imaging methods. It is therefore extremely useful for early diagnosis, particularly in high-risk patients who are on surveillance. EUS provides superior spatial resolution in detecting subtle changes in the parenchyma, tiny cystic lesions, and mural nodules within cysts. It is also well suited to evaluate vascular invasion, lymphadenopathy, and local resectability in suspected or proven cancer (81).

The incorporation of FNA provides the ability to acquire tissue samples for cytopathologic examination and molecular analysis, which are vital in distinguishing benign, pre-malignant, and malignant lesions (82). As an illustration, in assessing IPMNs, FNA can sample cyst fluid for tumor marker (e.g., Carcinoembryonic antigen (CEA)) and DNA mutation (e.g., KRAS, GNAS) assessment.

Molecular characterization assists in decision-making concerning operative intervention versus continued observation (83). In surveillance planning, especially for patients with FPC syndromes or identified precursor lesions, EUS is used in association with MRI/MRCP. This gives a non-invasive overview, and EUS is utilized when greater resolution or biopsy is required. The complementary use increases diagnostic sensitivity and can provide dynamic observation of changing lesions (81). Although it is useful, EUS is an invasive, sedation-requiring procedure that demands specialized skill. Complications are small but significant, including pancreatitis, hemorrhage, or infection, especially after FNA of cystic lesions. Thus, the choice to perform EUS with FNA is one of weighing diagnostic value against procedural risk, and it is usually reserved for situations where imaging is indeterminate or tissue confirmation is required.

CT with a dedicated pancreas protocol is widely employed imaging methodology to assess suspected PC. It has a primary role in initial diagnostic workup, staging, and surgical planning because it is broadly available, allows quick acquisition, and provides high spatial resolution (84). Nevertheless, even with these advantages, CT is generally less sensitive for the detection of early PC, particularly small lesions or cystic precursors, than more specialized imaging modalities such as MRI or EUS (85). Pancreas protocol CT includes multiphasic contrast-enhanced imaging, most commonly with arterial and portal venous phases, to maximize visibility of the pancreatic parenchyma and the adjacent vasculature. This is important in order to measure the tumor size, location, involvement of the vasculature, and the extent of metastatic disease, all of which are vital to the determination of resectability and treatment planning (86). CT can also accurately detect pancreatic ductal dilation, atrophy, and solid mass lesions, especially with more advanced disease (87). But CT is bedeviled by significant limitations for early detection and surveillance, particularly in high-risk patients or asymptomatic individuals. Minute lesions (<2 cm), initial PanIN, or minute ductal abnormalities can potentially be missed on CT because of compromised contrast resolution when compared with MRI or EUS (88). Also, CT does not have the capability to offer tissue sampling, an increasingly valuable tool for diagnosing indeterminate lesions and directing precision oncology strategies. In surveillance protocols, particularly in those at elevated genetic or family risk for PDAC, CT is not the first choice in imaging. Rather, practice guidelines from professional groups like the CAPS Consortium suggest MRI/MRCP and/or EUS, which are far more sensitive for small lesions and cystic abnormalities without subjecting patients to ionizing radiation. CT can still be employed as a follow-up imaging modality in certain situations, i.e., where MRI is contraindicated or to better assess the findings of other imaging modalities (89).

Positron Emission Tomography (PET) integrated with either CT (PET-CT) or MRI (PET-MRI) is a new modality actively being investigated for the imaging and surveillance of PC (90). These hybrid imaging modalities provide metabolic imaging abilities, which can supplement anatomical imaging by revealing regions of heightened glucose metabolism, a characteristic of most malignancies, including PDAC (91).

PET-CT, preferably done with 18F-fluorodeoxyglucose (18F-FDG), yields both metabolic and structural data, useful in the detection of occult metastases, evaluation of tumor response to treatment, and differentiation between benign and malignant lesions (92). Its ability for early-stage detection of PDAC, however, is limited at this time. This is so because small lesions or low-grade malignancies will have low FDG uptake, and inflammation or infection will cause false positives through high metabolic activity. In spite of these shortcomings, PET-CT has proved useful in staging, especially in the identification of distant metastases not detectable on CT or MRI (93).

PET-MRI is a newer, more sophisticated method that brings together the better soft-tissue contrast of MRI and the functional imaging of PET (94). The combination improves the capability to assess pancreatic lesions in detail, visualize early changes in tissue metabolism, and measure treatment response. PET-MRI also lowers radiation doses compared to PET-CT and is thus more appropriate for younger patients or those who need to have numerous follow-up scans. Nevertheless, its medical application is still limited for reasons of high expense, restricted availability, and the requirement for further confirmation in terms of large-scale studies (95).

Current recommendations do not yet support routine application of PET-based imaging for surveillance or the early detection of PDAC, particularly among asymptomatic high-risk individuals. Its main function currently is staging, restaging, and monitoring treatment, especially in instances where standard imaging is inconclusive. Current research is assessing the application of new PET tracers, such as those directed against specific molecular targets (e.g., integrins, fibroblast activation protein), that may enhance the specificity and sensitivity of PET in PC in the future. .

### 4.2 Comparative aspects

MRI/MRCP is best suited for non-invasive surveillance, EUS provides diagnostic depth and tissue sampling, CT is essential for staging and surgical planning, and PET-based imaging remains investigational. Together, these modalities form a complementary framework for early detection and management in high-risk populations shown in **Table 1**.

**Table 1:** Comparative framework of imaging modalities for high-risk pancreatic cancer patients.

Modality	Strengths	Limitations	Best-use cases	Surveillance suitability	Radiation	Operator dependence
MRI/MRCP (91)	Superior soft-tissue contrast; ductal mapping; no radiation	Cost, availability, contraindications	Cyst characterization, ductal disease, longitudinal surveillance	High – annual/biennial in high-risk cohorts	None	Moderate
EUS ± FNA (81, 84)	Highest spatial resolution; mural nodules; tissue acquisition (cytology, molecular)	Invasive; sedation; procedural risks	Indeterminate MRI/CT; suspected small lesions; cyst fluid analysis	Selective – adjunct to MRI/MRCP	None	High
CT (pancreatic protocol) (73, 74)	Widely available; rapid; vascular mapping; metastatic survey	Ionizing radiation; less sensitive for small/cystic lesions; no tissue sampling	Initial workup, staging, surgical planning	Low – not preferred for surveillance	Present	Low-Moderate
PET-CT/MRI (91)	Metabolic + anatomical imaging; staging; treatment monitoring	Cost; limited sensitivity for early disease; availability	Staging, restaging, treatment response	Investigational – not routine surveillance	PET-CT: Present; PET-MRI: Reduced	Moderate

### 4.3 Tiered approach to imaging in high-risk PC

Given the heterogeneity of pancreatic precursor lesions and the limitations of individual imaging modalities, contemporary surveillance strategies increasingly adopt a tiered approach. This framework prioritizes non-invasive, radiation-free techniques such as MRI/MRCP for baseline and longitudinal surveillance, while reserving EUS with FNA for situations requiring higher spatial resolution or tissue confirmation. CT remains indispensable for staging and surgical planning, whereas PET-based imaging is currently investigational, with potential future applications in precision oncology. Such a structured, stepwise integration of modalities ensures optimal sensitivity for early detection while balancing invasiveness, radiation exposure, and diagnostic yield. **Figure 2** illustrates the tiered strategy, highlighting how imaging choices are sequenced in the surveillance and diagnostic pathway for high-risk individuals.

**Figure 2:**
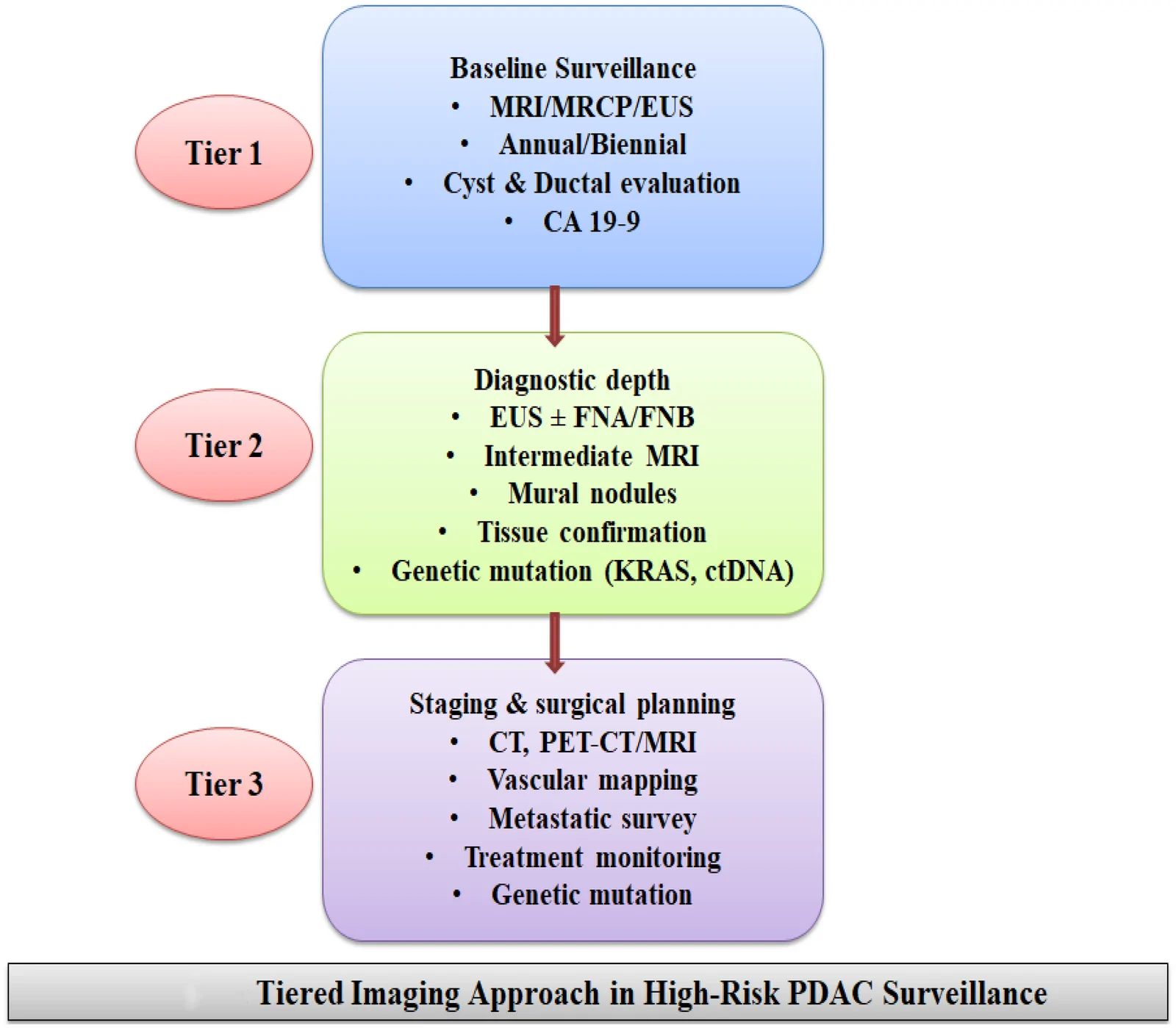
Tiered image approach in high-risk PDAC surveillance.

### 4.4 Serum biomarkers

CA 19–9 commonly used as a tumor marker in PC, but it has low sensitivity and specificity, especially for early detection (96). Although increased levels of CA 19–9 are observed in about 70-80% patients diagnosed with PDAC, this biomarker may also be elevated in other medical conditions which include cholangitis, pancreatitis, liver disease, and even in other gastrointestinal cancers (97). Due to such limitations, CA 19–9 is not appropriate to be used as a standalone screening method for asymptomatic individuals or for early detection of PDAC (98). Its sensitivity to detect early-stage tumors is low, thus many early cancers may not produce elevated level of CA 19-9. Furthermore, approximately 5-10% of the population is Lewis antigen-negative and does not produce CA 19-9, which results in false negatives (99). Yet, CA 19–9 is still useful as an additional diagnostic tool in symptomatic patients, such as those with jaundice, weight loss, or abdominal pain. Elevated levels of CA 19–9 may improve clinical concern and guide additional imaging and diagnostic testing. It is also useful for measuring treatment response and disease recurrence following surgery or chemotherapy (96).

## 5 Emerging biomarkers and liquid biopsy approaches

### 5.1 Circulating tumor DNA

ctDNA is a term used for DNA segments released into the bloodstream from apoptotic or necrotic tumor cells (100). In PC, ctDNA analysis has its potential as an ideal non-invasive biomarker for early detection, disease monitoring, and prognosis. Its aim is to identify tumor-specific genetic mutations without requiring invasive tissue biopsies (101). PDAC often carries mutations in important oncogenes and tumor suppressors, including KRAS, TP53, CDKN2A, and SMAD4 (102). Such mutations in plasma ctDNA can yield molecular proof of malignancy even at an early stage when tumor burden is low (103). Experiments have shown that detection of ctDNA is related to tumor burden and stage, and in some instances, can detect PDAC prior to radiographic findings (104).

Although promising, ctDNA analysis is hindered in early PDAC diagnosis by generally low levels of ctDNA released by small or localized tumors, necessitating sensitivity constraints (105). Emerging technologies like digital droplet PCR and next-generation sequencing have enhanced detection sensitivity and specificity, allowing for the detection of low-frequency mutations in plasma samples (106).

Notably, ctDNA also has the potential to address tumor heterogeneity and changing mutational profiles, possibly guiding targeted therapy. Blending ctDNA with other biomarkers (e.g., CA 19-9, protein panels) and imaging could improve early detection sensitivity.

### 5.2 Methylation DNA marker

DNA methylation, an epigenetic change that involves the addition of methyl groups to cytosine residues in CpG islands, is important for gene control and is commonly altered in cancer (107). Abnormal DNA methylation profiles, like hypermethylation of tumor suppressor gene promoters, are widespread in PDAC and may be used as sensitive biomarkers for early detection (108).

In PDAC, multiple genes show characteristic methylation alterations that distinguish malignant from benign or normal pancreatic tissue (109). For instance, promoter hypermethylation of the CDKN2A (p16), SPARC, TFPI2, and NPTX2 genes has been identified in tumor tissue from PC and cell-free DNA (cfDNA) in plasma or pancreatic juice (110). These methylation markers are potential candidates for early diagnosis because they can be identified non-invasively by liquid biopsy.

Identification of methylated DNA markers within plasma or pancreatic juice has been reported promising sensitivity and specificity in discriminating PDAC patients from controls as well as from pancreatitis individuals. The integration of panels of various methylation markers enhances diagnostic sensitivity, with potential to detect PDAC prior to the development of clinical symptoms (111).

Nonetheless, there are challenges in standardizing methods of assay, choosing the best marker panels, and validating results in large-scale prospective studies. Even with these challenges, methylated DNA markers hold a promising place for creating sensitive and specific early diagnostic tests for PDAC.

### 5.3 Protein- and metabolic biomarkers

Recent studies have looked at the application of protein panels consisting of tissue inhibitor of metalloproteinases 1 (TIMP1) and leucine-rich alpha-2-glycoprotein 1 (LRG1) in conjunction with CA 19–9 (112). TIMP1 is a glycoprotein that plays a role in extracellular matrix modulation and has been implicated in cancer development and adverse prognosis for PC (113). Increased blood levels of TIMP1 have been noted among patients with PDAC, and it has been suggested as a diagnostic marker (114). LRG1, in contrast, has functions in immune response and angiogenesis. Similar to TIMP1, LRG1 is also shown to be upregulated in PDACand is potentially used as a marker segregating malignant from benign pancreatic pathology (115).

Mixing these proteins with CA 19–9 has been found to enhance diagnostic performance considerably. For example, the panel consisting of TIMP1, LRG1, and CA 19–9 produced an area under the curve (AUC) greater than 0.9 for separation of PDAC from controls compared to using CA 19–9 alone (116). Such a panel might provide enhanced sensitivity in the early stages of disease when intervention becomes more likely to be curative. Furthermore, there is increased specificity that can lower false positives and prevent unwarranted invasive diagnostic tests (117).

The clinical significance of these results is significant. Protein panels like TIMP1 and LRG1 in combination with CA 19–9 may prove to be very valuable for screening high-risk individuals for PDAC, e.g., individuals with a strong family history or CP. Still, before such panels can be adopted into daily clinical practice, additional validation must occur in large, prospective, and ethnically heterogeneous cohorts. Standardization of assay techniques and cost analyses will also be essential to their successful implementation in real-world environments.

### 5.4 Exosomes and extracellular vesicles

Exosomes are tiny extracellular vesicles (30–150 nm) secreted by cells, including tumor cells, into the body fluids like blood, carrying proteins, lipids, RNA, and DNA that represent the molecular properties of their cells of origin (118). In PC, glypican-1 (GPC1)-positive exosomes have recently become a hopeful biomarker for early diagnosis (119).

GPC1 is a cell surface proteoglycan overexpressed on PC cells (120). GPC1+ exosomes can be extracted from serum of PDAC patients with high sensitivity and specificity, distinguishing them from healthy controls and patients with benign pancreatic disorders (121). Importantly, GPC1+ exosomes were found in the serum even in early-stage PDAC, potentially making them a useful biomarker (122).

These exosomes contain oncogenic proteins and nucleic acids, such as mutant KRAS and TP53 DNA, which give molecular information on tumor status without invading the tumor (123). Further, the content of GPC1+ exosomes was correlated with tumor burden and reduced following surgical resection, with implications for their potential use as indicators of disease progression and treatment response (119).

Even with these encouraging results, issues still exist with standardization of exosome isolation protocols and testing in larger, more diverse patient groups. More studies would be required to establish the clinical utility of GPC1+ exosomes as a standard biomarker for routine PDAC screening and early detection (122).

### 5.5 Blood-based assays

Blood is the most commonly utilized medium for liquid biopsy in the early diagnosis of PDAC, owing to its easy accessibility and the presence of various tumor-derived components (124). Several blood-based approaches have been developed and are under active investigation. One of the most prominent is the detection of ctDNA, which consists of small fragments of DNA released by tumor cells into the bloodstream. In PDAC, ctDNA analysis can reveal specific genetic mutations such as KRAS, TP53, and CDKN2A. Although the sensitivity of ctDNA is limited in early-stage disease, technological advances are steadily improving its detection capabilities (102).

Another important approach involves CTCs, which are intact cancer cells that have detached from the primary tumor and entered the bloodstream. These cells offer valuable insight into tumor biology and are being explored for their prognostic significance. Additionally, exosomes and other extracellular vesicles (EVs)—tiny, membrane-bound particles secreted by tumor cells carry a rich cargo of DNA, RNA, proteins, and lipids (125). Exosomes in particular have shown great promise in early PDAC detection, as they frequently contain tumor-specific mutations like KRAS (126).

Finally, blood-based protein biomarkers, such as CA 19-9, have long been used in clinical practice. However, CA 19–9 suffers from limited sensitivity and specificity, particularly for early-stage disease (127). To overcome these limitations, researchers are developing multi marker protein panels and combining them with ctDNA or exosome analysis to enhance diagnostic accuracy (128). Together, these blood-based strategies form a critical component of on-going efforts to detect PDAC at an earlier and more treatable stage.

### 5.6 Saliva, urine, and stool-based approaches

**Saliva biomarkers** are increasingly being seen as a new, non-invasive approach for the early diagnosis of PDAC (129). Saliva is a good choice of diagnostic fluid since it is easily collected, inexpensive, and amenable to large-scale screening. Saliva has traditionally not been linked to systemic diseases such as PC (130). However, recent literature has shown that systemic diseases could impact the salivary transcriptome and proteome, making saliva a promising medium to detect far-advanced malignancies (130).

The mechanisms by which PC affects salivary biomarkers have yet to be clearly defined, but exosomes—small extracellular vesicles secreted by tumor cells—are thought to be the key players (131). Tumor-secreted exosomes may pass through the circulatory system to the salivary glands and release their molecular payload, such as RNA and proteins, into saliva. This inter-organ communication allows the systemic disease to impart a detectable molecular print on saliva, even though there is physical distance between the pancreas and oral cavity.

Saliva diagnostics may be particularly useful in resource-poor environments or for population-level screening, when invasive or costly testing is impractical. For patients at increased risk of PDAC—e.g., those with familial risk, genetic syndromes (e.g., BRCA mutation), or onset of diabetes—the use of saliva testing could offer a first-line screening test (132). Additional research will be required to confirm these results in larger, prospective populations and to optimize collection and analysis protocols.

Urine-based biomarkers have been developed as a novel, non-invasive method for the early detection of PDAC (133). In comparison with blood-based markers, urine has a number of advantages: it is simple to collect, repeated sampling is possible, and it has simpler protein content, making it an ideal medium for the detection of disease-specific markers (134). Since current blood-based biomarkers such as CA 19–9 have limitations, urine biomarkers are increasingly being explored for their ability to identify PC at an earlier, more curable stage (135).

A number of these urinary proteins and metabolites have been discussed in recent research as having potential in identifying early PDAC. One good example includes a panel made up of LYVE1 (lymphatic vessel endothelial hyaluronan receptor 1), REG1A (regenerating islet- derived 1 alpha), and TFF1 (trefoil factor 1). In a seminal study, this panel was found to efficiently discriminate between early-stage PDAC and healthy controls and benign pancreatic disorders. An AUC of 0.89 was reported, which increased to 0.94 when CA 19–9 was included in the panel, showing the potential for improved diagnostic discrimination when applied in combination (136). LYVE1 is implicated in lymphangiogenesis and is considered to be an indication of tumor-induced lymphatic alterations (137). REG1A is implicated in regeneration of pancreatic tissue and has been reported elevated in CP and PDAC, yet measurements are generally higher in malignancy (138). TFF1 is implicated in mucosal protection and repair, and its overexpression has been correlated with many cancers, including PDAC (139). The combination of the three markers creates a good signature that can be used to distinguish malignant from non-malignant diseases.

Urine tests with such panels may be especially valuable among high-risk groups, like those with a family history of PC, recent-onset diabetes after age 50, or CP (140). These non- invasive analyses may be used as screening tests first; identifying individuals who need to be further investigated using imaging or biopsy. Nevertheless, in spite of early promising results, these results need to be corroborated in multicenter large clinical trials before they can be implemented generally in clinical practice.

Stool-based approaches are emerging as a non-invasive and promising strategy for the early detection of PDAC. The rationale behind stool testing lies in the anatomical proximity of the pancreas to the gastrointestinal tract, allowing for the potential shedding of tumor-derived materials such as DNA, RNA, proteins, or even whole cells into the stool (141). Researchers have found that certain genetic mutations commonly associated with PDAC, particularly KRAS mutations, can be detected in stool samples. This is especially valuable for identifying early-stage or precancerous lesions before clinical symptoms appear.

In addition to direct genetic alterations, changes in the gut microbiome have also been linked to PDAC. Studies have shown that patients with pancreatic tumors often exhibit unique microbial profiles in their stool compared to healthy individuals. These microbial signatures could serve as indirect biomarkers, enhancing the sensitivity and specificity of stool-based diagnostics (142).

Another advantage of stool-based testing is its convenience and acceptability for patients, making it suitable for large-scale screening, especially in high-risk populations. While this approach has been more widely applied to colorectal cancer, on-going research is adapting and refining stool-based assays for PDAC detection. Techniques such as next-generation sequencing (NGS) and digital PCR are being utilized to improve the accuracy of detecting low-abundance tumor markers in stool (143).

However, challenges remain. The sensitivity of detecting PDAC-specific markers in stool can be limited due to the dilution and degradation of tumor DNA in the gastrointestinal tract (144). Moreover, distinguishing PC signals from those arising from other GI conditions requires highly specific biomarker panels. Despite these hurdles, stool-based diagnostics hold considerable potential as part of a multi-modal screening strategy aimed at catching PC at its earliest, most treatable stages.

## 6 Multi-omics integration for early diagnosis

The term “multi-omics” refers to the comprehensive integration of various biological data types that reflect different levels of cellular activity and regulation. These include genomics, which involves the study of DNA sequences and mutations; transcriptomics, which focuses on the analysis of RNA expression; proteomics, which examines the profiling of proteins and their functional roles; and metabolomics, which investigates the small molecules, or metabolites, produced during cellular processes (145).

These individual layers of biological information are not independent but rather interconnected, each offering a unique perspective on tumor behavior. When analyzed collectively, they provide a holistic view of tumor development, progression, and molecular complexity. This integrated approach is especially valuable in the context of PDAC—a highly aggressive and biologically heterogeneous cancer—where single-layer data often fail to capture the full picture of disease onset and evolution (146). Multi-omics integration, therefore, holds significant promise for improving early detection, diagnosis, and personalized treatment strategies in PDAC.

### 6.1 Genomics + proteomics: complementary diagnostic layers

In PDAC, genomic alterations are frequent and well-characterized. Common driver mutations include KRAS (90–95%), TP53 (50–75%), CDKN2A (30–50%), and SMAD4 (30–35%). These mutations can be detected in ctDNA found in blood samples (147).

However, while ctDNA can indicate the presence of cancer, its sensitivity in early-stage disease remains suboptimal due to the low abundance of DNA fragments shed into the bloodstream (148). To overcome this, genomic data are often combined with proteomic biomarkers—such as CA 19-9, CEA, or newer markers like THBS2 (thrombospondin-2). Studies have shown that integrating ctDNA with proteomic data significantly enhances early detection sensitivity. For example, a 2018 study by Cohen et al. reported that a combined ctDNA and protein marker panel could detect early-stage PDAC with sensitivity of 64% and specificity of 99.5%—a major improvement over traditional tests (149).

### 6.2 Transcriptomics: capturing gene expression changes

Transcriptomics focuses on RNA molecules, especially mRNA, that reflects active gene expression in cells. In cancer, transcriptomic changes often precede detectable alterations in DNA or proteins, making RNA analysis a sensitive early detection tool (150). In the context of liquid biopsy, RNA sequencing (RNA-seq) of exosomes or CTCs can uncover oncogenic transcriptional activity. For example, overexpression of genes related to epithelial-mesenchymal transition (EMT), angiogenesis, or immune evasion may be identified through transcriptomics, even when tumor burden is minimal (151).

Moreover, non-coding RNAs like microRNAs (miRNAs) and long non-coding RNAs (lncRNAs) found in plasma or exosomes have emerged as powerful early biomarkers. For instance, miR-21, miR-155, and miR-196a have all been reported to be overexpressed in early PC and precursor lesions (e.g., PanINs, IPMNs) (152).

### 6.3 Metabolomics: revealing tumor metabolism

PDAC is known for its altered metabolism, which supports rapid cell growth in a nutrient-poor microenvironment. Metabolomics analyses the small molecules (like amino acids, lipids, and sugars) produced or consumed by cancer cells (153).

Tumor metabolism leaves a distinct chemical signature that can be detected in blood, urine, or saliva. For example, pancreatic tumors show elevated levels of metabolites such as glycolytic intermediates, branched-chain amino acids (BCAAs), and lipid derivatives (154). A study published in Nature (2014) identified a panel of BCAAs that could predict PC up to two years before diagnosis (155).

When metabolomics is combined with genomic or proteomic data, it can reveal how genetic mutations affect metabolic pathways, providing deeper insight into tumor activity. This multi-layer integration significantly boosts diagnostic power.

### 6.4 Machine learning integration: turning big data into actionable insights

The integration of multi-omics data generates vast, complex datasets. Extracting meaningful diagnostic insights from these data requires advanced computational tools, particularly machine learning (ML) and artificial intelligence (AI) (156).

AI algorithms can be trained on multi-omics datasets to recognize patterns associated with early-stage PDAC. These models can weigh the relative contribution of various markers— mutations, RNA expression, protein levels, and metabolic changes—and create a composite risk score or prediction model (157).

In recent studies, AI models using multi-omics inputs have achieved AUC values > 0.90, indicating very high diagnostic accuracy (156). For example, AI-driven models combining ctDNA mutations, miRNA profiles, and metabolomics have been able to differentiate early PDAC from benign conditions like CP with high sensitivity and specificity.

## 7 Role of non-coding RNAs in early diagnosis

ncRNAs are RNA molecules that do not encode proteins but play critical regulatory roles in gene expression and cellular function. In recent years, ncRNAs have emerged as important molecular players in cancer biology, including PDAC). Their stability in body fluids, such as blood, saliva, and urine, along with their disease-specific expression patterns, make them highly promising biomarkers for early diagnosis (158).

### 7.1 MicroRNAs (e.g., miR-21, miR-155)

MicroRNAs (miRNAs) are small RNA molecules with non-coding function that modulate gene expression after transcription and exert important functions in cancer development and progression (159). In PDAC, certain miRNAs, including miR-21 and miR-155, have been recognized as new biomarkers for early diagnosis because of their aberrant expression in tumor tissues and in the circulation (160). Research has indicated that miR-21 is commonly overexpressed in PC cells and patient serum and enhances tumor proliferation, invasion, and apoptosis resistance (161). Likewise, miR-155 is overexpressed in PC and participates in inflammatory processes as well as tumor advancement (162). The miRNAs are detectable in blood plasma, serum, and even pancreatic juice, presenting a minimally invasive method of detecting pancreatic malignancy (163).

Notably, miR-21 and miR-155 levels in the circulation can distinguish PC patients from healthy controls as well as patients with benign pancreatic diseases like CP, with good diagnostic capability (164). In combination with other biomarkers such as CA 19-9, miRNA panels enhance the sensitivity and specificity of early detection of PDAC (165).

Obstacles still exist to standardizing miRNA detection methods and validating the biomarkers in large, prospective clinical trials. However, recent evidence points towards miRNAs as promising non-invasive, early diagnostic markers that may complement current screening measures to enhance care for PDAC patients.

## 8 Artificial intelligence and machine learning in early diagnosis

ML and AI are fast reshaping the face of PDAC detection and risk estimation by boosting the functions of imaging modalities and fusing enormous quantities of clinic data for predictive analytics. These technologies present promising pathways for earlier and more precise diagnosis of PDAC, especially in asymptomatic or high-risk individuals (166).

### 8.1 Imaging-based AI

Conventional imaging techniques like CT, MRI, and EUS tend to miss early PC because precursor lesions are small and present subtly. AI and ML-driven algorithms are being generated to improve image analysis, recognizing patterns and features that may not be discernible to the human eye. For instance, large data-trained deep learning models are able to detect subtle parenchymal alterations, ductal abnormalities, or early atrophy or fibrosis changes that occur before PDAC develops (167).

Current research has shown that AI-augmented radiologic interpretation can enhance the detection of tumors early on, particularly lesions less than 2 cm in size (168). Convolutional neural networks (CNNs), a type of deep learning highly adapted to image recognition, have been used on both CT and MRI scans to determine pancreatic masses with high accuracy and specificity. In a few instances, AI software surpassed even skilled radiologists at detecting early-stage lesions or determining risk of progression in cystic lesions such as IPMNs (78).

Additionally, AI systems are being piloted to automate pancreatic tissue segmentation, normalize reporting, and combine imaging with other clinical data to develop risk scores or decision support results that can assist clinicians in making better surveillance and treatment decisions.

## 9 Prediction models using electronic health records

Concurrently with advances in imaging, AI and ML are also being used to extract EHR) data to predict risk. Such models integrate large numbers of variables—demographic variables, laboratory testing, comorbidities, medications, and longitudinal health data—to identify patients who should undergo additional pancreatic assessment (169).

One prominent example is the ENDPAC score, a clinical risk model that uses EHR data to stratify patients with NOD, a known early manifestation of PC. The model incorporates factors such as age, weight loss, and glycemic variability to identify individuals at elevated risk for PDAC, guiding them toward further testing like MRI or EUS (170).

Beyond ENDPAC, on-going research aims to integrate multi-modal datasets—including genomics, proteomics, and lifestyle factors—into ML frameworks to develop more comprehensive and personalized risk models. Some algorithms are already capable of flagging high-risk individuals up to 6–12 months before a clinical diagnosis, opening a critical window for early intervention (171).

## 10 Pancreatic cyst surveillance programs

Pancreatic cysts are increasingly found because of the common use of cross-sectional imaging, and it is necessary to stratify these cysts accurately according to their risk of malignancy for management. The important features related to malignancy are cyst size, mural nodules, and main pancreatic duct (MPD) dilation. Cysts over 3 cm in size, enhancing mural nodules, and an MPD diameter of ≥10 mm are known as high-risk features. Those of concern are 2–3 cm cysts, mural nodules not enhancing, and between 5–9 mm dilation of the MPD. Low-risk cysts are normally symptomatic, smaller than 2 cm, and without nodules or ductal change (85).

Management of pancreatic cysts is by the Fukuoka and American Gastroenterological Association (AGA) guidelines. According to the Fukuoka guidelines, surgery by resection should be done for cysts with high-risk stigmata such as enhancing mural nodules or major MPD dilation (172). For cysts with worrisome characteristics and EUS with or without FNA should be used to further assess the risk, as per the guidelines. Without concerning features, surveillance is recommended according to cyst size and patient factors like age and comorbidities (85). The AGA guidelines are more liberal, however, with a recommendation of EUS-FNA only in the case of two or more high-risk features, including cyst size ≥3 cm, solid component, or duct dilation. Surgery is only recommended if concerning findings are confirmed by EUS or cytology (173).

EUS and cyst fluid analysis are critically important in the risk stratification of indeterminate pancreatic cysts. CEA measurement in the fluid is beneficial in identifying mucinous cysts; values greater than 192 ng/mL are predictive of a mucinous origin (174, 175). Cytology provides a definitive diagnosis with the presence of malignant cells, but its sensitivity is poor. Molecular markers increasingly are being utilized to enhance diagnostic yield. KRAS and GNAS mutations are typical for endogenous IPMNs, whereas TP53, PIK3CA, and PTEN mutations could be associated with high-grade dysplasia or invasive carcinoma (176).

Methods for early diagnosis of PDAC seek to detect malignancy at a potentially curable stage, most importantly in patients with high-risk pancreatic cysts. Surveillance using MRI, CT, or EUS is utilized depending on the degree of risk ascertained by imaging and clinical presentation (177). EUS-FNA with sophisticated cytopathological evaluation enhances sensitivity for detecting early malignancy. Molecular cyst fluid analysis and novel liquid biopsy methods have potential for non-invasive early detection. In addition, the use of artificial intelligence and radiomics for imaging interpretation is being explored to improve malignancy prediction in cystic lesions (178). These strategies, along with guideline-driven management, provide a systematic approach to early detection and treatment of PDAC.

## 11 Conclusions

PDAC is still dismally diagnosed at a late, non-resectable stage. This review highlights that successful early detection will need a multimodal risk-adapted approach combining the clinical risk factors, identification of precursor lesions, advanced imaging and novel biomarkers. MRI/MRCP has emerged as the preferred non-invasive modality for screening, especially in relation to PCLs and ductal changes, and EUS offers high-resolution imaging with potential for tissue and molecular analysis when combined with FNA. Clinical conditions such as NOD and CP offer important diagnostic windows for targeted evaluation. Collectively, these strategies highlight the need to move beyond symptom-based diagnosis toward proactive, evidence-informed early detection to improve resectability and survival in PDAC.

## 12 Limitations

Despite advances, several limitations persist. Most evidence supporting early detection strategies is derived from observational studies or high-risk cohorts, limiting applicability to the general population. Current imaging modalities cannot reliably predict which precursor lesions will progress to invasive disease, raising concerns regarding over-surveillance and overtreatment. Invasive techniques such as EUS are resource-intensive and unsuitable for large-scale screening. Moreover, many promising biomarkers lack standardized validation and remain investigational, with limited integration into routine clinical practice. Health system constraints, including cost, accessibility, and specialist availability, further hinder widespread implementation of early detection strategies.

## 13 Future perspective

Future progress in early PDAC diagnosis will depend on integrated, precision-based strategies that combine clinical risk profiling with molecular and imaging data. Blood-based biomarkers, including ctDNA, exosomal RNA, and metabolic signatures, hold promise for non-invasive screening and longitudinal monitoring. AI and machine-learning tools applied to radiomics and EHR may enhance early lesion detection and refine risk prediction. Prospective, multicenter trials are essential to validate these approaches, establish standardized surveillance algorithms, and demonstrate clinical benefit. Ultimately, a multidisciplinary and translational framework will be critical to achieving earlier diagnosis and improving outcomes in PDAC.
